# Does tumor-infiltrating lymphocyte therapy improve survival outcomes in patients with advanced melanoma?

**DOI:** 10.3389/fmed.2026.1775031

**Published:** 2026-02-04

**Authors:** Piper Ridley-Parish

**Affiliations:** School of Medicine, Swansea University, Swansea, United Kingdom

**Keywords:** advanced melanoma, checkpoint inhibitors, CTLA4, IL-2, metastatic melanoma, tumor-infiltrating lymphocyte therapy

## Abstract

Metastatic melanoma is an aggressive form of cancer, with poor patient outcomes when first-line treatments fail. Success has been seen in checkpoint blockade immunotherapies such as anti-PD-1 and anti-CTLA4 treatments, however long-term use results in resistance. Tumor-infiltrating lymphocyte (TIL) therapy is effective in treating melanoma as a second-line option, particularly in cancers such as melanoma, breast and ovarian cancer. With the 2024 FDA approval of Iifileucel (Amtagvi), a type of TIL therapy, this literature review aims to establish how effectively TIL therapy can treat metastatic and advanced melanomas, to evaluate if this type of therapy should be approved in the UK. A detailed search of databases Medline and Cochrane Library was conducted using terms related to “tumor-infiltrating lymphocyte therapy” and “advanced melanoma” focusing on peer-reviewed research and phase II/III clinical trials published between 2015 and 2025. Studies were included if written in English and reported survival-related outcomes. The evidence highlights TIL therapy as an effective treatment for late-stage melanoma, with up to 20% of patients showing complete responses and all studies regarding TIL therapy as equally effective or more successful than the control drugs used. However, some studies reported adverse effects, mostly linked to lymphodepleting chemotherapy. Overall, the literature indicates that TIL therapy is a breakthrough drug that could change the treatment of melanoma, and allow patients another, effective treatment option, after first-line treatments are no longer suitable. This review concludes that future research should focus on larger cohort studies, and cost-effectiveness investigations to support approval in the UK.

## Introduction

Advanced melanoma is one of the most aggressive malignancies, with poor prognoses once checkpoint inhibitors (ICIs) or targeted therapies fail. Checkpoint blockade immunotherapy (CBI) has been the first-line treatment for metastatic melanoma, with success seen in anti-programmed cell death protein 1 (anti-PD-1) treatment and anti-cytotoxic T-lymphocyte antigen 4 (anti-CTLA4) therapy. Long-term use leads to resistance and disease progression, highlighting the urgent need for second-line defensive treatments (1, 2). Tumor-infiltrating lymphocyte (TIL) therapy is an adoptive cell transfer approach which uses the patient's T-cells to recognize specific tumor antigens and target cancer cells. The therapy has shown promise in melanoma, breast, ovarian, and other cancers (2, 3).

Early meta-analyses highlighted TIL's clinical benefit in heavily pretreated patients, with objective response rates (ORR) around 41% and complete response rates (CRR) approximately 14% (3). Recent research confirms ongoing efficacy in patients after CBI resistance (1). Despite this, TIL therapy is linked to treatment-related adverse effects involving lymphodepleting chemotherapy and high-dose interleukin-2 (IL-2) (4). Analyses found TILs to be cost-saving and clinically beneficial when compared with treatments like ipilimumab (5). TIL therapy is viewed as an evidence-based treatment with potential to reinvent management of advanced melanoma.

In 2024, the US FDA approved Iifileucel (Amtagvi) for advanced melanoma (6). As the first TIL therapy authorized, this marked a breakthrough in oncological research (6, 7). However, in the UK, evaluations by NICE are still ongoing (ID3863) (8). highlighting the need to evaluate survival outcomes to support future approval and clinical use of TIL therapy.

Therefore, the purpose of this review is to compare TIL therapy with current/standard treatment to determine TILs efficacy for managing advanced melanoma.

## Search strategy and selection criteria

A systematic literature review was conducted using databases Medline and Cochrane Library to gather high-quality medical and clinical articles. To establish a clear and focused research question, the PICO framework was used, illustrated in Supplementary Table 1.

From this, initial searches for “tumor-infiltrated lymphocyte therapy” and “advanced melanoma” were performed to ensure enough research was available. Next, Boolean operators were incorporated to narrow the search to relevant literature. Supplementary Table 2 shows search terms used and the number of articles that were found at the time of the search.

Inclusion criteria:

- English language
- Peer-reviewed
- Human adults (≥18 years)
- Reporting survival-related outcomes

The reference lists were downloaded and added to Endnote 2025 to remove duplicates.

**Figure 1:**
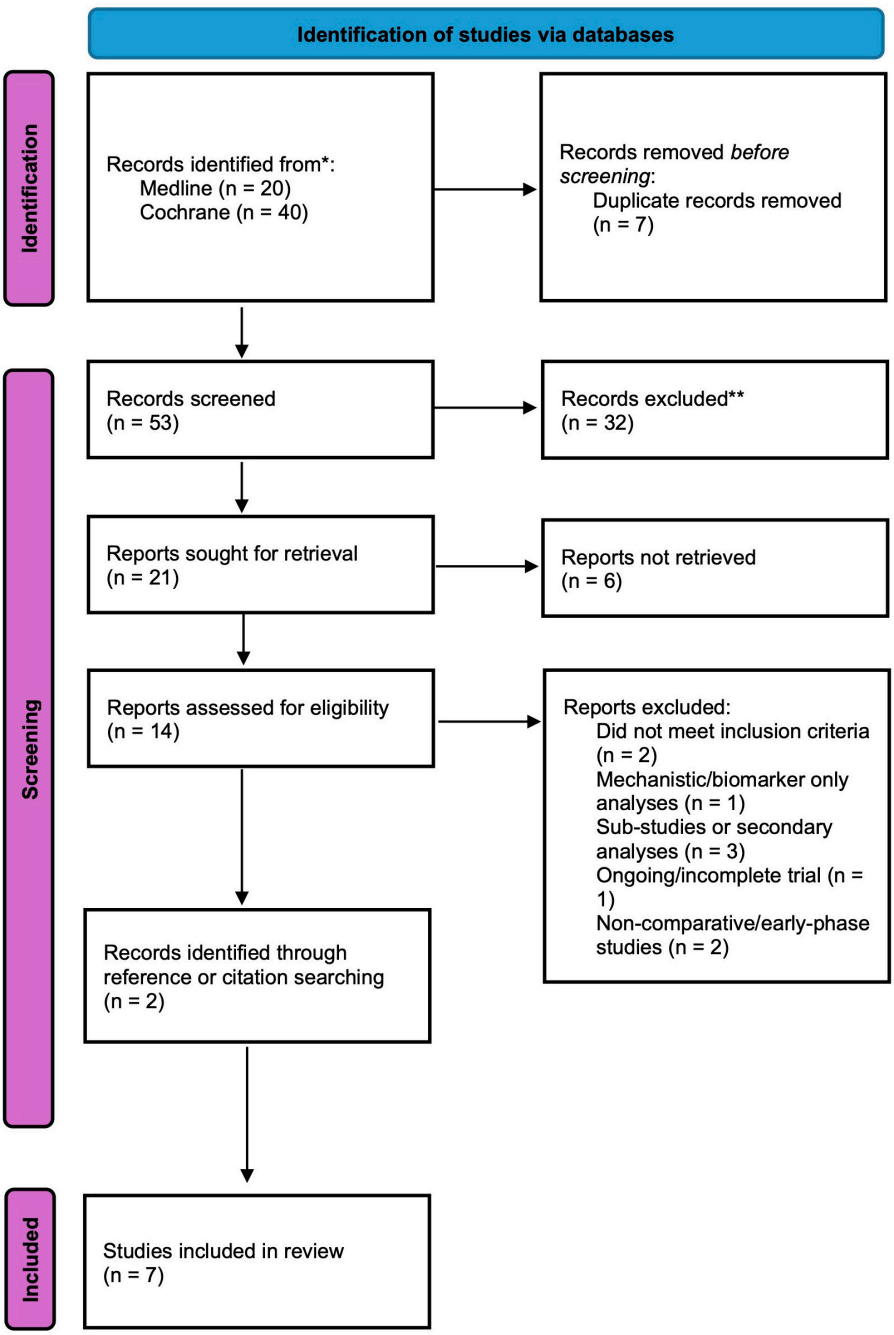
PRISMA flow diagram to illustrate the process of selecting appropriate articles. The diagram outlines the identification, screening, eligibility, and inclusion of studies.

Seven studies were found to fit inclusion criteria. A breakdown of the selection process is presented in Figure 1.

Eligible studies included randomized controlled trials, phase II/III clinical trials, and prospective cohort studies directly evaluating TIL therapy. Data on study design, sample size, intervention details, and survival outcomes were compared across the seven included studies.

## Findings

The characteristics/main findings of the seven included studies are summarized in Supplementary Table 3. Studies evaluated adoptive cell therapy using tumor-infiltrating lymphocytes (TIL-ACT) in advanced melanoma, with variations in methodology.

### Andersen et al. (9)

- Fifteen females and 10 males.
- Overall response rate (ORR) = 42%, with three complete responses (CR) and seven partial responses (PR).
- Median overall survival (OS) of 21.8 months.
- More infused tumor-reactive T cells = better tumor regression.
- Toxicities were observed (IL-2 related) but manageable without ICU involvement.

### Goff et al. (10)

- Female CR 24%, PR 32%.
- Male CR 24%, PR 28%.
- *P* = 0.54 for sex difference.
- CRR were 24% in both groups.
- OS was 38.2 (TBI) vs. 36.6 months (no TBI; *p* = 0.71).
- Thirteen of 48 patients in the TBI arm reported Thrombotic microangiopathy, with this adverse event absent from non-TBI patients.
- Median survival was over 3 years.

### Hasanov et al. (11)

- Fifty seven percent males, 43% females.
- ORR was 14%.
- One “Arm 2” patient showed PR for over 76 months.
- Median OS was 9.7 months in high-dose IL-2, and 8.8 months in low-dose.

### Rohaan et al. (12)

- Patients tested were 60% male, 40% female.
- In TIL group: 56% male, 44% female.
- Ipilimumab group: 63% male, 37% female.
- Median progress-free survival was 7.2 months in the TIL group, and 3.1 months in ipilimumab group.
- ORR = 49% in TIL, and 21% in ipilimumab.
- Adverse events recorded in all who received TIL therapy, and 57% of ipilimumab patients.

### Saberian et al. (13)

- Nine females and nine males overall.
- TIL group: four females, six males.
- TIL + DC: five females, three males.
- ORR = 39% (30% in TIL + 50% in TIL + DC).
- OS duration was 4.1 years in TIL and 2.0 years in TIL + DC (*p* = 0.47).

### Forget et al. (14)

- Twenty seven females, 47 males.
- ORR was 42%: 47% no prior checkpoint inhibitors, 38% anti-CTLA4 therapy, and 33% both anti-CTLA4 and anti-PD1 therapy.
- Median survival was 17.3 months, highest (24.6 months) for patients with no prior CTLA4 treatment.
- Patients who received more TILs had better outcomes, only with no prior ant-CTLA4 therapy.

### Medlina et al. (7)

- Overall, 54.2% males, 45.8% females.
- ORR was 31.4% (5.9% CR, 25.5% PR).
- Median duration of response was 36.5 months.
- OS of 13.9 months (5-year OS 19.7%).
- Seventy nine percent showed tumor shrinkage.
- Adverse effects consistent with lymphodepletion/IL-2 therapy.

## Discussion

Across the studies, ORR ranged from 14 to 49% and OS ranged from 8 to 48 months, showing consistent survival benefit compared to controls. All studies showed TIL-ACT produce CRs, observed in up to 20% of patients, suggesting long-term remission is achieved in certain individuals.

The included studies had several strengths. Most were phase II/III randomized controlled trials with populations of refractory stage III–IV melanoma (10, 12). Long-term follow up data confirmed that TIL therapy shows sustained responses (7). Interventions across the studies were generally standardized, with the majority using lymphodepletion chemotherapy and IL-2, which improved comparability studies.

Limitations, such as small sample sizes were present, particularly in early studies (9, 13). Studies also varied in IL-2 dosing and extra treatment inclusion e.g., vaccines or pembrolizumab. Toxicity was not always isolated to chemotherapy or IL-2, so could be directly linked to TIL therapy (4).

All included studies reported gender-based distributions of patient cohorts. The majority demonstrated balanced sex representation. Across all trials, no statistically significant differences were reported in treatment response between sexes, e.g., one study reported no association between sex and outcome (*P* = 0.54) (10). In contrast, a recent analysis identified a significant association between sex and progression-free survival (*P* = 0.029), suggesting a potential female survival advantage with TIL therapy (15). However, this finding was derived from a small cohort (seven males, six females), limiting the generalizability of the result and therefore this observation should be validated through larger clinical studies.

Although study designs differed, all supported TIL-ACT as an effective treatment for advanced melanoma. Studies showed improved progression-free survival when compared with ipilimumab and lasting benefits after Iifileucel infusions (7, 12). Evidence suggests that TIL therapy can improve survival outcomes, but treatment doses and supportive care need further refinement to reduce toxicity.

In conclusion, research supports TIL therapy as a second-line treatment for advanced melanoma, offering long-term benefits. However, larger cohort studies, such as TILVANCE-301 (16), and cost-effectiveness investigations (5) are needed to confirm benefits and support approval in the UK.
