# Supplementary material for: Does tumor-infiltrating lymphocyte therapy improve survival outcomes in patients with advanced melanoma?
Source: Front Med (Lausanne). 2026 Feb 4;13:1775031. doi: 10.3389/fmed.2026.1775031 (PMC12913130; doi:10.3389/fmed.2026.1775031)
Supplement: Supplementary file 1 [file Supplementary_file_1.docx]

**Supplementary Material**

1. **Supplementary Tables**
   1. **Supplementary Table 1.** PICO strategy to identify research areas to form the review question.

| Population | Adults with advanced or metastatic melanoma |
| --- | --- |
| Intervention | Treatment with tumour-infiltrating lymphocyte (TIL) therapy |
| Control | Standard treatments such as checkpoint inhibitors |
| Outcome | Improved survival outcomes, including progression-free survival and overall survival |

- 1. **Supplementary Table 2.** Search strategy used across both Medline and Cochrane Library.

| Database searched | Search terms used | Articles found |
| --- | --- | --- |
| Medline | TX ("tumour-infiltrating lymphocyte*" OR "tumor-infiltrating lymphocyte*" OR "TIL therapy")  AND TX ("advanced melanoma" OR "metastatic melanoma")  AND TX ("overall survival" OR "progression-free survival" OR "treatment outcome" OR efficacy) | 20 |
| Cochrane | ("tumour-infiltrating lymphocyte*" OR "tumor-infiltrating lymphocyte*" OR "TIL therapy")  AND ("advanced melanoma" OR "metastatic melanoma")  AND ("overall survival" OR "progression-free survival" OR "treatment outcome" OR efficacy) | 40 |

- 1. **Supplementary Table 3.** Summary of included studies evaluating TIL therapy outcomes in advanced melanoma.

| Author & Year | Study Design | Sample Size | Intervention Details | Comparator | Main Survival Outcomes | Key Findings/Notes |
| --- | --- | --- | --- | --- | --- | --- |
| Andersen et al., 2016 | Phase I/II clinical trial (single-arm, open label) | 25 patients with treatment-refractory metastatic melanoma | TIL-ACT following lymphodepleting chemotherapy (cyclophosphamide + fludarabine) and attenuated continuous decrescendo IL-2 regimen instead of high-dose bolus IL-2 | None (single-arm trial; compared with historical HD-IL-2 TIL data in discussion) | - ORR = 42% (3 CR + 7 PR) - Median PFS = 3.9 months (NR group = 3.3 months; responders not reached) - Median OS = 21.8 months (NR = 13.1 months; responders not reached) - 1-year OS = 72%, 3-year OS = 40.8% | - Demonstrated durable complete responses with reduced IL-2 toxicity. - Toxicities manageable without ICU support. - Antitumour T-cell persistence correlated with response. - Suggests high-dose IL-2 not essential for efficacy, supporting broader clinical feasibility of TIL-ACT. - 15 females and 10 males - No sex-response association commented on |
| Goff et al., 2016 | Randomised phase II clinical trial | 101 patients with metastatic melanoma (76 with M1c disease) | TIL-ACT following nonmyeloablative chemotherapy (cyclophosphamide + fludarabine) with or without 1,200 cGy total body irradiation (TBI), followed by high-dose IL-2 | NMA chemotherapy alone vs NMA + TBI | - CR rate: 24% in both arms - Median OS: 38.2 months (TBI) vs 36.6 months (no TBI) - Median PFS: 9.6 vs 7.5 months (no significant difference) - 3-year OS = 51% overall | - Addition of TBI did not improve survival or response rates, but increased toxicity (notably thrombotic microangiopathy). - Demonstrated durable complete respnses and long-term survival in around 24% of patients. - Confirms that TIL-ACT alone is sufficient for durable remission in metastatic melanoma without intensified lymphodepletion. - Female CR (24%), PR 12 (32%), NR 16. Men CR 15 (24%), PR 18 (28%), NR 31 - P = .54 for sex difference |
| Hasanov et al., 2024 | Randomised phase II clinical trial | 14 patients with metastatic melanoma (most stage IV M1c/d; heavily pre-treated) | TIL-ACT after lymphodepletion (cyclophosphamide + fludarabine) with high-dose IL-2 (Arm 1) or low-dose IL-2 (Arm 2), followed by pembrolizumab every 3 weeks | High-dose IL-2 vs low-dose IL-2 post-TIL, both with pembrolizumab | - ORR = 14% overall (1 PR in each arm) - Median PFS: 3.9 mo (HD) vs 2.1 mo (LD) - Median OS: 9.7 mo (HD) vs 8.8 mo (LD) - One Arm-2 patient had ongoing PR > 76 months | - No significant survival or response differences between IL-2 doses. - Low-dose IL-2 showed similar efficacy with reduced toxicity (fewer febrile neutropenia events, shorter hospital stay). - Pembrolizumab did not enhance response in previously ICI-treated patients. - Suggests low-dose IL-2 may be a safer, viable alternative in TIL therapy without compromising efficacy. - 57% males, 43% females. - No sex-response distributions commented on. |
| Rohaan et al., 2022 | Phase 3 multicentre randomised controlled trial | 168 patients with unresectable stage IIIC-IV advanced melanoma (86% refractory to anti-PD-1 therapy) | TIL-ACT following lymphodepleting chemotherapy (cyclophosphamide + fludrabine) and high-dose IL-2; compared to ipilimumab 3 mg/kg every 3 weeks x 4 doses | Ipilimumab monotherapy | - Median PFS: 7.2 mo (TIL) vs 3.1 mo (ipilimumab) - HR 0.50 (95% CI 0.35-0.72; P<0.001) - Median OS: 25.8 mo (TIL) vs 18.9 mo (ipilimumab) - ORR: 49% (TIL) vs 21% (ipilimumab) - CR rate: 20% (TIL) vs 7% (ipilimumab) | - First phase 3 evidence showing TIL therapy significantly improves PFS and ORR vs standard immunotherapy. - Durable complete responses and better quality-of-life scores in TIL group despite higher toxicity. - Confirms TIL-ACT as an effective second-line option for advanced melanoma refractory to PD-1 inhibitors. - Male 100 patients (60%). Female 68 patients (40%). - Til group: 56% male, 44% female. Ipilimumab group: 63% male, 37% female. - No sex-response association commented on. |
| Saberian et al., 2021 | Randomised phase II clinical trial | 18 treated patients (10 received TIL alone; 8 received TIL + dendritic cell vaccine) | TIL-ACT following non-myeloablative lymphodepleting chemotherapy and high-dose IL-2, with or without MART-1 peptide-pulsed dendritic cell (DC) vaccination | TIL + DC vaccine vs TIL alone | - ORR: 39% overall (30% TIL vs 50% TIL + DC) - Median PFS: 0.26 years (TIL) vs 0.49 yrs (TIL + DC) - Median OS: 4.1 yrs (TIL) vs 2.0 yrs (TIL + DC) - 2-yr OS: 58% (TIL) vs 50% (TIL + DC) - One TIL + DC patient had ongoing CR > 10 years | - No significant improvement in TIL persistence or survival with DC vaccination. - TIL + DC arm showed numerically higher ORR, but likely due to higher infused TIL counts. - Confirms durable long-term remission possible with TIL therapy alone. - Suggests limited benefit of single-antigen (MART-1) DC vaccines; multi-antigen or radiation combinations may offer more promise. - Female 9, Male 9. - TIL alone: 4F/6M. TIL + DC: 5F/3M. - No sex-response distributions commented on. |
| Forget et al., 2018 | Prospective single-centre phase II analysis (MD, Anderson, USA) | 74 patients with stage IIIC-IV metastatic melanoma (43 checkpoint-naïve; 30 previously exposed to anti-CTLA4 +- anti-PD1) | Autologous TIL-ACT following lymphodepleting chemotherapy (cyclophosphamide + fludarabine) and high-dose IL-2; all received 1-2 IL-2 cycles post-infusion | Checkpoint-naïve vs anti-CTLA4-exposed cohorts | - Overall ORR: 42% (11% CR, 31% PR) - ORR by prior therapy: 47% (naïve) vs 33-38% (anti-CTLA4 +- PD1) - Median PFS: 4.0 mo - Median OS: 17.3 mo (24.6 mo naïve vs 8.6 mo anti-CTLA4; HR 2.3, p = 0.003) - 1-yr OS: 58%, 2-yr OS: 40%, with durable CRs >7 years | - Prior anti-CTLA4 exposure reduced TIL efficacy, shortening OS and duration of response. - High TIL count, CD8+ proportion, and BTLA expession correlated with response – but only in checkpoint-naïve patients. - Tumour mutation load and recognition unaffected by prior therapy. - Baseline serum IL-9 > 5.3 pg/mL predicted response – first reported blood biomarker for TIL-ACT. - Confirms that TIL therapy remains effective post-checkpoint blockade, though responses are less durable. - 27 females, 47 males - Sex was not identified as a significant predictor of overall survival. |
| Medlina et al., 2025 | Phase II multicentre single-arm trial (C-144-01; 5-year follow-up) | 153 treated patients with unresectable/metastatic melanoma refractory to anti-PD-1/PD-L1 +- BRAF/MEK inhibitors | Single infusion of lifileucel TIL therapy following non-myeloablative lymphodepletion (cyclophosphamide + fludarabine) and <6 doses of high-dose IL-2 | None (single-arm study; long-term follow-up analysis) | - ORR: 31.4% (CR 5.9%, PR 25.5%) - Median DOR: 36.5 mo (95% CI, 8.3-NR) - Median OS: 13.9 mo (95% CI, 10.6-17,8) - 5-year OS: 19.7% - 5-year ongoing response: 31.3% of responders - Median time to response: 1.4 mo | - Demonstates durable long-term efficacy of one-time lifileucel in heavily pretreated, checkpoint-refractory melanoma. - 79% of patients had tumour shrinkage; 4 PR->CR conversions >1 year post-infusion. - Toxicity conistent with NMA-LD/IL-2, resolving within 2 weeks; no new long-term safety signals. - Confirms lifileucel provides a meaningful long-term survival benefit and supports its use post-PD-1/BRAF/MEK therapy. - Male 83 (54.2%). Female (45.8%). - No sex-response distributions commented on. |
